# Prediction of myopia onset and shift in premyopic school-aged children: a machine learning-based algorithm

**DOI:** 10.3389/fmed.2025.1646277

**Published:** 2025-11-17

**Authors:** Mingjun Gao, Yanhua Hou, Yutong Lu, Zhanhua Shi, Qi Zhao

**Affiliations:** Department of Ophthalmology, The Second Affiliated Hospital of Dalian Medical University, Dalian, China

**Keywords:** premyopia, myopic progression, subfoveal choroidal thickness, machine learning, prediction model

## Abstract

**Purpose:**

This study aimed to investigate longitudinal changes in ocular parameters and develop a machine learning-based model for predicting myopia onset and shift within 1 year in school-aged premyopic children.

**Methods:**

This prospective cohort study enrolled 320 premyopic children aged 6–12 years from the Ophthalmology Clinic of The Second Affiliated Hospital of Dalian Medical University. Uncorrected visual acuity (logMAR), cycloplegic spherical equivalent (SE), axial length (AL), average corneal curvature (CC), and subfoveal choroidal thickness (SFCT) were measured at baseline and 6-month intervals for 12 months. Premyopia was defined as - 0.50 D < SE ≤ + 0.75 D. A multivariable analysis evaluated predictive factors including age, gender, parental myopia, baseline SE, AL, CC, axial length/corneal radius (AL/CR), and SFCT. Machine learning algorithms were used to predict 1-year myopia onset and myopia shift, along with Shapley Additive exPlanations (SHAP) interpretation.

**Results:**

Among 284 participants (88.8% retention rate), 141 children (49.3%) developed myopia. The cohort exhibited an annual SE progression of −0.695 ± 0.222 D and AL elongation of 0.356 ± 0.122 mm. The AL/CR increased from 2.986 ± 0.061 to 3.029 ± 0.072 (*p* < 0.001), while SFCT demonstrated a significant reduction of 21.535 ± 9.731 μm (*p* < 0.001). The optimal model achieved an AUC-ROC of 0.963 (95% CI: 0.930–0.997) for myopia onset prediction, with baseline SE emerging as the most significant predictor, followed by parental myopia, SFCT, and age. Meanwhile, our algorithm also achieved clinically acceptable 1-year predictions of SE.

**Conclusion:**

Premyopic children exhibited accelerated myopic progression. Our machine learning-based predictive models showed promising performance for myopia onset and myopia shift, providing clinically valuable risk stratification for targeted prevention strategies.

## 1 Introduction

Myopia has become a major global public health issue, especially in East Asia. Among Chinese children and adolescents, its prevalence shows an annual increase, increasing from 55.5% in 2010 to 60.1% in 2019, with the peak age of onset dropping from 12 years old in 2010 to 7 years old in 2019 (1). An earlier onset of myopia is associated with a higher risk of developing more severe myopia in adulthood. The rapid increase in high myopia cases, coupled with population aging, suggests a potential dramatic increase in vision-impaired pathologic myopia over the coming decades (2), underscoring the critical importance of predicting the risk of myopia and implementing early interventions.

To enable early intervention in children at imminent risk of myopia onset, the International Myopia Institute (IMI) introduced the concept of “premyopia” in 2019. This condition is defined as a refractive state with a spherical equivalent between −0.50 D and +0.75 D. When combined with baseline refraction, age, and other quantifiable risk factors, these factors indicate a sufficient likelihood of future myopia development to warrant preventive intervention (3). This definition has been consistently adopted in the IMI white papers on myopia management in 2021 and 2023 (4, 5).

Premyopia represents a substantial proportion of children. The Ireland Eye Study (IES) reported a premyopia prevalence of 32.4% among schoolchildren aged 6–7 years (6), while a Spanish study documented a prevalence of 42.4% among children aged 5–7 years (7). In China, a study conducted in Shanghai found a premyopia prevalence of 21.9% among school-aged children, which significantly exceeded the prevalence of myopia (2.8%) (8). In Taiwan, the prevalence of premyopia reached 52% among preschool children (9). Children in the premyopic phase often exhibit no significant visual impairment and lack subjective complaints, leading to frequent oversight by their parents.

A previous study has shown that the axial length of the eye grows rapidly before myopia onset (10). Furthermore, the rate of myopic shift and axial elongation in the year preceding myopia onset exceeds that observed in children with myopia (11). Therefore, premyopia represents a critical period characterized by rapid axial elongation and heightened myopia risk, while also constituting a vital window for management and early intervention.

Although established methods effectively control myopia progression, they carry potential side effects. The necessity of early clinical intervention for all premyopic cases remains uncertain. Consequently, identifying individuals at the highest risk who warrant early treatment is clinically imperative. Rational and effective premyopia management could shift the focus of myopia control earlier, thereby mitigating myopia onset and progression.

Machine learning (ML), an automated approach to data analysis for model building, surpasses traditional linear regression by effectively managing complex non-linear relationships and delivering superior predictive performance. In ophthalmology, ML is applied in areas such as disease diagnosis, severity grading, and progression prediction (12). Notable examples include its role in diagnosing diabetic retinopathy (13), predicting myopia progression (14, 15), customizing contact lens parameters (16, 17), and forecasting visual acuity outcomes following treatment for neovascular age-related macular degeneration (18). Various ML algorithms are available, each with unique strengths and limitations. Random forest, a supervised learning algorithm, is extensively used in classification and regression tasks and serves as the foundation of many modern machine learning systems. Although there is a tendency for overfitting, this risk can be minimized by careful system design.

Therefore, this prospective observational study aims to investigate the characteristics of myopia progression in school-aged premyopic children. Leveraging baseline clinical data and ML techniques, the study further aims to predict myopia onset and myopic shift in this population, identify potential risk factors for myopia development, and accurately stratify high-risk premyopic children to guide clinical interventions.

## 2 Methods

### 2.1 Study population

This prospective longitudinal observational study enrolled premyopic children aged 6–12 years at the Ophthalmology Clinic of The Second Affiliated Hospital of Dalian Medical University between September 2023 and March 2024. The study protocol was approved by the Ethics Committee of The Second Hospital of Dalian Medical University and adhered to the tenets of the Declaration of Helsinki. Written informed consent was obtained from the parents or legal guardians of all participants.

### 2.2 Sample size

Based on clinical relevance, model generalizability, and measurement feasibility, nine variables—gender, age, parental myopia, and standard ophthalmic parameters—were included, with an events-per-variable (EPV) ratio set at 10. Based on prior literature indicating a 1-year myopia incidence rate of approximately one-third in premyopia (19), a total of 320 subjects were recruited, accounting for a 15% attrition rate and the requirements for model accuracy.

### 2.3 Inclusion and exclusion criteria

All participants met the following inclusion criteria: children aged 6–12 years with cycloplegic spherical equivalent refraction ≤ + 0.75 D and > − 0.50 D in both eyes, astigmatism or anisometropia of −1.00 D or less in both eyes, best-corrected distance visual acuity of 0.20 logMAR or better in both eyes, intraocular pressure (IOP) of less than 21 mmHg, and legal guardians who fully comprehended the study and provided signed informed consent. The exclusion criteria were as follows: children with other ocular diseases (e.g., strabismus, amblyopia, cataract, other media opacities, or ocular tumors), previous or current treatment with myopia control interventions (e.g., atropine and multifocal spectacles), inability to cooperate with ophthalmic examinations, or inability to complete two follow-up visits due to geographical constraints.

### 2.4 Study procedures

No clinical intervention was performed. Standard myopia prevention education was provided during visits, including limiting continuous near work to ≤40 min per session and ensuring ≥10 h of weekly outdoor sunlight exposure. Baseline and 6- and 12-month follow-up data included sex, age, parental myopia history, uncorrected visual acuity (UCVA), cycloplegic spherical equivalent refraction (SE), axial length (AL), subfoveal choroidal thickness (SFCT), average corneal curvature (CC), and axial length-to-corneal radius ratio (AL/CR). These parameters were analyzed to develop prediction models for 1-year SE progression and myopia onset.

Children exhibiting UCVA >logMAR 0.2 in both eyes during follow-up were prescribed single-vision spectacles for classroom use. SE was calculated as the sphere plus half of the cylindrical power. Myopia was defined as SE ≤ −0.50 D.

SE was measured three times using an autorefractor (ARK-1, NIDEK, Japan), with the average calculated. All 3 readings should be at most 0.25D apart in both the spherical and cylinder components. Three drops of 1% cyclopentolate (Alcon) were instilled at 5-min intervals. Refraction was performed 30 min after the last drop. A fourth drop was administered if the pupillary light reflexes persisted or pupil size was <6.0 mm 15 min post-instillation.

CC and AL were measured three times using the Lenstar LS900 (Haag-Streit, Switzerland), and averages were recorded. AL/CR = AL (mm) × mean CC (D)/337.5.

UCVA was measured using a logMAR chart (VSK-VC-Y; WeiShiKang, Guangzhou, China).

SFCT was measured vertically from Bruch’s membrane to the choroid–scleral interface at the foveal center using OCT (Cirrus HD-OCT 5000; Carl Zeiss, Germany). OCT imaging was conducted at similar time points to minimize diurnal variation. All scans were acquired without any cycloplegia.

### 2.5 Statistical analysis

Statistical analyses were performed using SPSS (version 27.0) and Python statsmodels (version 0.13.2). Continuous variables were analyzed using independent samples t-tests and Wilcoxon rank-sum tests, while categorical variables were assessed using chi-squared tests. Within-group changes were evaluated using repeated-measures analysis of variance (ANOVA). A *p*-value of < 0.05 was deemed statistically significant.

### 2.6 Algorithm design

We developed a machine learning-based binary classification model to predict 1-year myopia incidence and a regression model to predict SE at follow-up, using clinically collected metrics. The dataset was split into 70% for training and 30% for testing using a stratified random sampling approach. Stratification was based on the outcome variable (myopia onset) to ensure a similar distribution of events between the sets. This split ratio is conventional in machine learning and was deemed adequate given the number of features and events, thereby helping to mitigate overfitting while maintaining sufficient power for validation.

#### 2.6.1 Classification model development and evaluation

Univariate and multivariate logistic regression analyses were used to find significant clinical predictors of myopia onset. Features with a *p*-value of < 0.05 in the multivariate analysis were incorporated into machine learning algorithms. Five algorithms, Logistic Regression (LR), Naïve Bayes (NB), Decision Tree (DT), Random Forest (RF), and Support Vector Machine (SVM), were comprehensively evaluated to optimize myopia-onset prediction performance. The diverse algorithmic spectrum ensured robust capture of complex data patterns. We used a 5-fold cross-validation (cv. = 5) combined with a grid search (GridSearchCV) technique to identify the optimal hyperparameters and prevent overfitting. The best set of hyperparameters identified through the above cross-validation process was used to train a final model on the entire 70% training set. This final model was then evaluated on the completely unseen 30% test set. Test-set performance metrics included accuracy, precision, recall, F1-score, and area under the receiver operating characteristic curve (AUC) (20). We combined Shapley Additive exPlanations (SHAP) feature importance with correlation coefficients to visualize each feature’s predictive contribution.

#### 2.6.2 Regression model development and evaluation

Random Forest Regressor was used for regression tasks. Hyperparameters were optimized via grid search (GridSearchCV) to enhance predictive performance. Features were scaled to [0, 1] using min–max normalization to improve the model efficacy. Five-fold cross-validation (cv. = 5) mitigated overfitting, and parallel processing (n_jobs = −1) accelerated the computations.

The optimized model was trained on the training set. The model performance was quantified using the mean squared error (MSE), mean absolute error (MAE), root mean squared error (RMSE), and coefficient of determination (R^2^) between the predicted and actual SE values across the training and test sets, ensuring generalizability.

## 3 Results

### 3.1 Baseline characteristics of premyopic children

Table 1 presents the baseline characteristics of the study cohort. A total of 284 school-aged premyopic children completed the 1-year follow-up (attrition rate: 11.3%).

**Table 1:** Characteristics of premyopia children who were included in the study at baseline (*n* = 284).

Variable	ALL	6–<8 years	8–<10 years	10–<12 years	*P*
Sex, *n* (%)					0.015
Female	147 (51.761)	70 (58.824)	56 (47.863)	21 (43.750)	
Male	137 (48.239)	49 (41.176)	61 (52.137)	27 (56.250)	
Parental myopia, *n* (%)					0.008
0	26 (9.155)	7 (5.882)	18 (15.385)	1 (2.083)	
1	114 (40.141)	55 (46.218)	36 (30.769)	23 (47.917)	
2	144 (50.704)	57 (47.899)	63 (53.846)	24 (50.000)	
UCVA (logMAR)	0.004 ± 0.051	0.014 ± 0.048	0.008 ± 0.052	−0.028 ± 0.043	<0.001
SE (D)	0.332 ± 0.313	0.468 ± 0.264	0.235 ± 0.310	0.229 ± 0.313	<0.001
AL (mm)	23.135 ± 0.676	22.814 ± 0.647	23.324 ± 0.573	23.470 ± 0.653	<0.001
CC (D)	43.585 ± 1.363	43.839 ± 1.319	43.470 ± 1.331	43.237 ± 1.456	0.017
AL/CR	2.986 ± 0.061	2.961 ± 0.052	3.002 ± 0.058	3.005 ± 0.068	<0.001
SFCT (μm)	307.768 ± 34.464	315.958 ± 34.254	309.378 ± 36.535	302.769 ± 31.763	0.066

UCVA = uncorrected visual acuity; SE spherical equivalent; AL = axial length; CC = corneal curvature; AL/CR = axial length/corneal radius; SFCT = subfoveal choroidal thickness.

### 3.2 Myopia progression in Premyopic children over 1 year

After 1 year, 141 children developed myopia (incidence rate: 49.3%), with the rate being 51.7% (76/147) among female and 47.4% (65/137) among male children. The mean annual refractive progression was −0.695 ± 0.222 D, with a mean axial elongation of 0.356 ± 0.122 mm. The corneal curvature did not show any significant changes. The AL/CR increased from 2.986 ± 0.061 at baseline to 3.029 ± 0.072. SFCT decreased significantly by 21.535 ± 9.731 μm during the follow-up period (Table 2).

**Table 2:** Changes in ocular parameters during the follow-up stage.

Variable	Baseline	6 months	12 months	*P*-value
UCVA (logMAR)	0.004 ± 0.051	0.030 ± 0.080	0.096 ± 0.116	<0.001
SE (D)	0.332 ± 0.313	0.038 ± 0.349	−0.363 ± 0.401	<0.001
AL (mm)	23.135 ± 0.676	23.305 ± 0.674	23.492 ± 0.681	<0.001
CC (D)	43.585 ± 1.363	43.591 ± 1.379	43.583 ± 1.360	0.997
AL/CR	2.986 ± 0.061	3.001 ± 0.075	3.029 ± 0.072	<0.001
SFCT (μm)	307.768 ± 34.464	295.884 ± 33.494	286.232 ± 33.937	<0.001

UCVA = uncorrected visual acuity; SE spherical equivalent; AL = axial length; CC = corneal curvature; AL/CR = axial length/corneal radius; SFCT = subfoveal choroidal thickness.

### 3.3 Myopia onset prediction

There was no statistically significant difference in the baseline characteristics between the test and training sets, ensuring no bias between the groups (Supplementary Table 1). Univariate and multivariate logistic regression analyses identified significant features in the training set. Features with a *p*-value of < 0.05 in the multivariate analysis—baseline SE, SFCT, age, and parental myopia—were incorporated into the machine learning prediction models (Supplementary Table 2).

**Figure 1:**
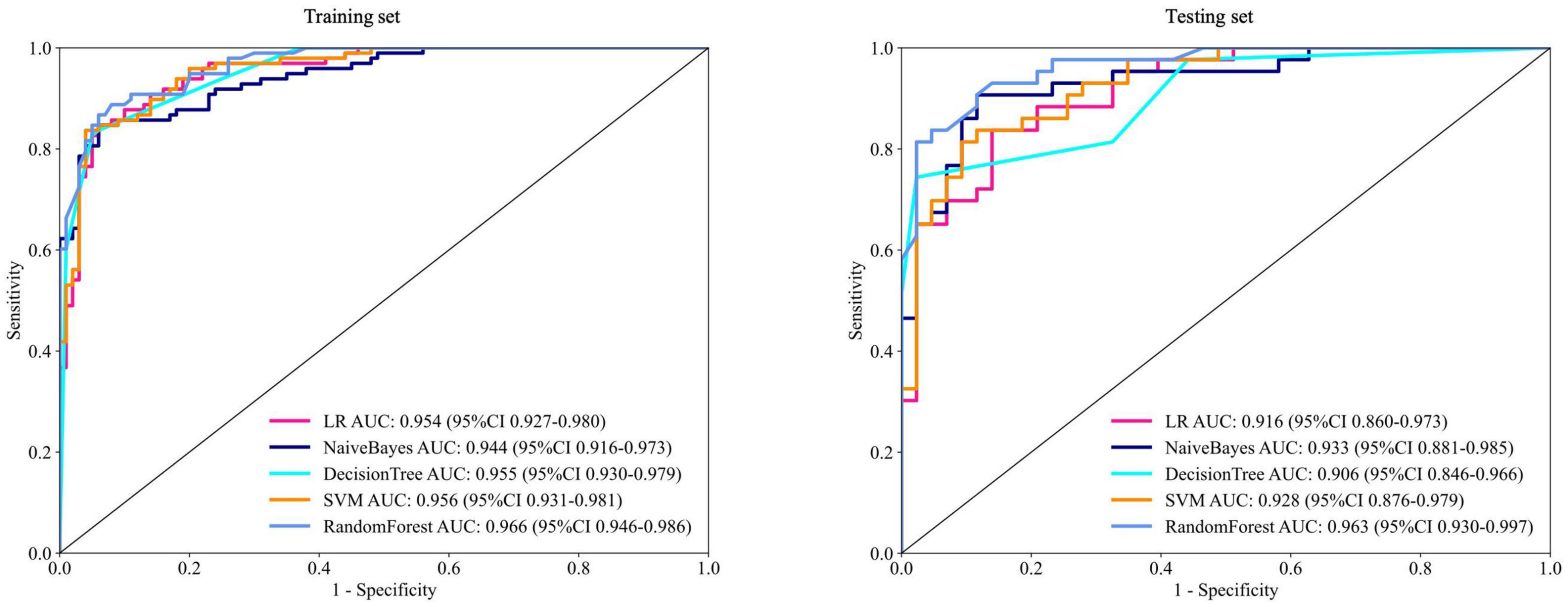
ROC of five machine learning algorithms for predicting the risk of myopia onset at 12 months in the training and test sets. ROC = receiver operating characteristic curve; AUC = area under the receiver operating characteristic curve.

The performance metrics of the five clinical models were compared. The DT underperformed on the test set, particularly in terms of sensitivity and recall, suggesting potential overfitting. LR and NB showed consistent performance across the training and test sets, though AUC and accuracy were slightly lower in the test set. SVM demonstrated strong training performance but a modest decline in testing. RF achieved a high AUC with balanced precision and recall in both sets (Figure 1 and Table 3).

**Table 3:** Performance metrics of different models on the training set and test set.

Cohort	Model	AUC	AUC 95%CI	Acc	Acc 95%CI	Sen	Spe	PPV	NPV	Precision	Recall	F1 score	Cutoff
Train	LR	0.954	0.9274–0.9801	0.889	0.8451–0.9327	0.837	0.940	0.932	0.855	0.932	0.837	0.882	5.870e-01
Test	LR	0.916	0.8598–0.9726	0.826	0.7337–0.8962	0.791	0.860	0.848	0.806	0.848	0.791	0.819	5.870e-01
Train	NB	0.944	0.9156–0.9732	0.889	0.8392–0.9285	0.837	0.940	0.932	0.855	0.932	0.837	0.882	6.545e-01
Test	NB	0.933	0.8810–0.9849	0.860	0.8053–0.9147	0.814	0.907	0.897	0.830	0.897	0.814	0.853	6.545e-01
Train	DT	0.955	0.9303–0.9795	0.889	0.8451–0.9327	0.827	0.950	0.942	0.848	0.942	0.827	0.880	5.051e-01
Test	DT	0.906	0.8455–0.9662	0.860	0.7684–0.9248	0.791	0.930	0.915	0.815	0.915	0.791	0.849	5.051e-01
Train	RF	0.966	0.9458–0.9860	0.899	0.8570–0.9410	0.867	0.930	0.924	0.877	0.924	0.867	0.895	4.983e-01
Test	RF	0.963	0.9303–0.9966	0.872	0.8215–0.9225	0.814	0.930	0.919	0.826	0.919	0.814	0.863	4.983e-01
Train	SVM	0.956	0.9305–0.9811	0.894	0.8333–0.9242	0.827	0.960	0.953	0.850	0.953	0.827	0.885	6.894e-01
Test	SVM	0.928	0.8756–0.9794	0.837	0.7429–0.9055	0.767	0.907	0.886	0.802	0.886	0.767	0.822	6.894e-01

LR = Logistic Regression; NB = Naïve Bayes; DT = Decision Tree; RF = Random Forest; SVM = Support Vector Machine; Acc = accuracy; Sen = sensitivity; Spe = specificity; PPV = positive predictive value; NPV = negative predictive value.

**Figure 2:**
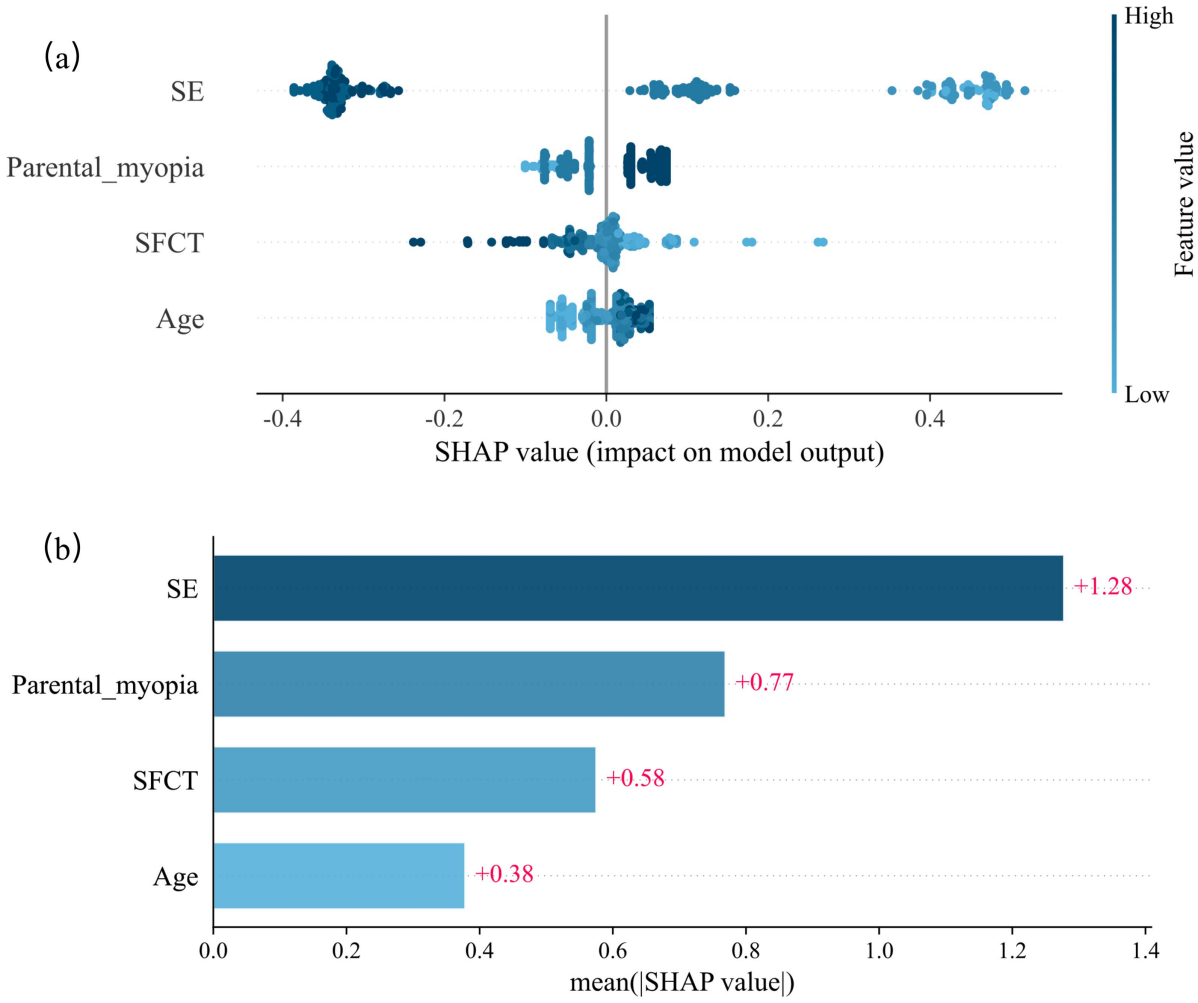
SHAP values of the random forest model for predicting the risk of myopia onset at 12 months. **(a)** Scatter plot of SHAP values of the random forest model. **(b)** The average SHAP values of the model output by the four indicators. SHAP = Shapley Additive exPlanations.

Shapley Additive exPlanations (SHAP) analysis was used to quantify feature contributions to the predictions. Lower baseline SE, parental myopia, thinner SFCT, and older age increased the 1-year myopia risk. Baseline SE exerted the strongest influence on the model output, followed by parental myopia, SFCT, and age (Figure 2).

### 3.4 Myopia progression prediction

**Figure 3:**
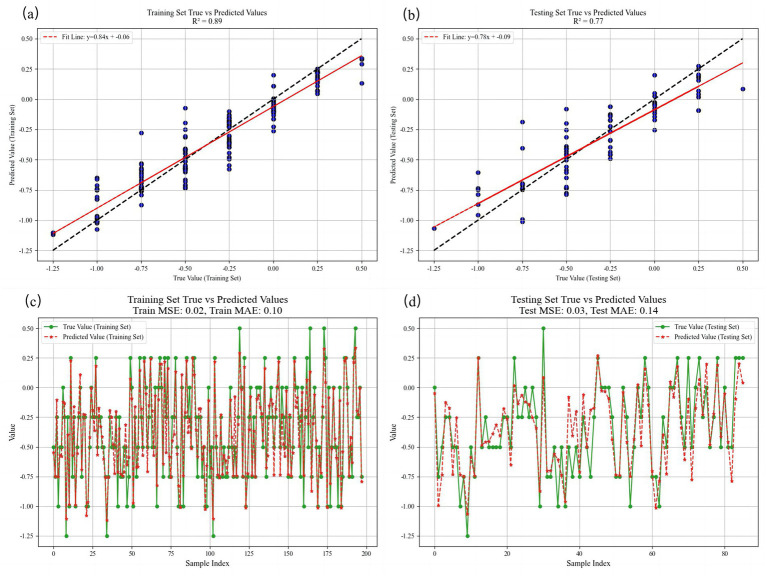
Comparison of SE predicted values and true values at 12 months in both training and testing sets. **(a)** The fitting situation of linear regression in the training set. **(b)** The fitting situation of linear regression in the testing set. **(c)** Visualization of the comparison between the true value and the predicted value in the training set. **(d)** Visualization of the comparison between the true value and the predicted value in the testing set. R2 = coefficient of determination, MSE = mean squared error, MAE = mean absolute error, and RMSE = root mean squared error.

The trained model achieved an *R*^2^ of 0.8931 in the training set, indicating an excellent data fit. The MAE, MSE, and RMSE of the training set were 0.1002, 0.0179, and 0.1337, respectively, indicating that the model had good predictive performance on the training set. For the test set, *R*^2^ = 0.7667, MAE = 0.1417, MSE = 0.0335, and RMSE = 0.1830. Despite the slightly reduced test performance, the prediction accuracy remained robust (Figure 3).

## 4 Discussion

Previous machine learning-based models primarily focused on diagnosing non-cycloplegic myopia and predicting risks of high myopia or pathologic myopia (8, 14, 21, 22), with insufficient consideration for early intervention in premyopia. Our study provided the longitudinal evidence establishing the predictive value of ocular and genetic factors for myopia development in premyopic children. Using the predictive modeling approach, children identified as high risk for developing myopia within 1 year may be considered for early interventions such as low-concentration atropine eye drops (23), peripheral defocus spectacles (24), or a combination thereof. Children at low risk may avoid unnecessary interventions beyond reinforced lifestyle education. This approach holds significant potential for delaying myopia onset and advancing personalized treatment.

Premyopic individuals exhibit depleted hyperopic reserve and elevated myopia risk (25, 26). A previous study reported that approximately one-third of premyopic children developed myopia within 1 year, compared to a < 1% incidence among non-premyopic emmetropic children (19). Children with baseline SE > +2.00 D showed a 5-year myopia incidence of merely 4.4% versus 92.0% among those with SE between 0.00 and −0.50 D (25). Our findings confirmed this high-risk profile, demonstrating a 49.3% 1-year myopia incidence in school-aged premyopes. Therefore, our study was designed to capture the initial onset and shift in myopia within a high-risk premyopic population. The 1-year horizon allowed us to observe early changes and build a foundational model.

We further identified that lower baseline SE, parental myopia, thinner baseline SFCT, and older age increased the risk of myopia. Baseline SE emerged as the strongest predictor of myopia onset within 1 year among premyopic children in China, with lower SE values conferring a higher risk. Although AL is significant in myopia control (27), the baseline AL and AL/CR ratio were not predictive in our study. This discrepancy may stem from variations in the AL-SE correspondence. Our cohort showed a mean annual AL increase of 0.356 ± 0.122 mm, with 1 mm AL elongation corresponding to 1.95 D SE progression, consistent with previous reports that found SE changes of 0.83 D, 1.74 D, and 1.83 D per 1 mm AL increase in emmetropes, premyopes, and myopes, respectively (28). This gradient is reflected in lens power loss (29). During abnormal axial elongation, the crystalline lens may initially compensate for the myopic shift; however, this compensatory mechanism diminishes at myopia onset (30), exacerbating SE progression per millimeter of AL growth. Consequently, SE changes outpaced axial elongation in premyopia. Additionally, lens power decreased with age (31), which aligns with our observation of a higher myopia incidence in older children, suggesting lens involvement in myopiogenesis.

The choroid, a highly vascularized structure that nourishes the retinal pigment epithelium and outer retina, is critical for retinal function (32). The IMI discussed choroidal involvement in myopia in the latest series of myopia control (33). Choroidal thinning, which is strongly correlated with AL elongation, may coincide with myopia onset. Animal studies have indicated that chicks with initially thinner choroids exhibit faster axial growth than those with thicker choroids (34). However, clinical evidence establishing a thinner SFCT as a predictor of subsequent myopia in premyopic children remains unreported. Our study addressed this gap, demonstrating a significant SFCT reduction (21.535 ± 9.731 μm) during follow-up. Predictive modeling further established a thinner baseline SFCT as a significant risk factor for 1-year myopia development in premyopia.

While our algorithm demonstrated promising predictive performance with the ocular parameters and parental myopia history, a previous study found that gender differences (particularly higher susceptibility in females), urban–rural residence disparities, and reduced outdoor activity time were also significant independent risk factors affecting the occurrence of myopia. Their study documented a distinct risk hierarchy: urban female > urban male > rural female > rural male children. These factors likely operate through multiple pathways, including differential educational pressures, variations in natural light exposure affecting dopamine-mediated ocular growth regulation, and gender-specific hormonal influences on scleral remodeling (35).

Although the incidence of myopia was slightly higher in female (51.7%) than in male children (47.4%) in our cohort, gender did not demonstrate significant predictive value during feature selection for the prediction model. Tideman et al. (36) reported that risk scores combining environmental factors and ocular parameters could identify high-risk children. Another model suggested that ocular factors outweighed environmental and genetic predictors in myopia progression (22). Another cohort study found that 2-year myopia incidence was correlated solely with parental myopia, independent of environmental factors such as near-work duration, diopter hours, outdoor time, or tutoring (28). Environmental factors (e.g., daily near-work duration, outdoor activity, and lighting conditions) present high measurement challenges and significant quantification errors due to behavioral inconsistencies and seasonal variations. Consequently, to ensure model robustness, these factors were excluded from the prediction algorithm and addressed through post-prediction clinical counseling. Our algorithm exclusively focuses on stable, quantifiable predictors (e.g., biometric parameters, baseline refractive status, and genetic markers).

## 5 Limitations

This study had several limitations. First, this single-arm cohort study lacked a control group, preventing comparison of ocular progression in children with different refractive statuses. Considering the current effectiveness of myopia control strategies, we assessed the ethical justification for establishing an untreated myopia control group. Additionally, due to the necessity and practical difficulties of clinical cycloplegic examinations, no high hyperopia reserve control group was included. Consequently, the model is best suited to risk stratification within a premyopic cohort identified by current clinical standards rather than for screening the general pediatric population. This design choice, while pragmatic, may restrict the model’s utility in settings where population-wide screening is required.

Second, the sample size was constrained by single-center recruitment, which precluded external validation and may limit the generalizability of the findings. Although internal validation was conducted, external testing is required before clinical implementation. Moreover, the algorithm was developed and validated exclusively for Chinese school-aged children (6–12 years). While core biometric relationships suggest cross-population applicability, clinical use in non-Asian cohorts will require local calibration and prospective validation. Finally, due to the high incidence of premyopia converting into myopia within 1 year, our model only predicted the risk of myopia occurrence within a specific 12-month period. Given the single-center design, limited follow-up period, and lack of external validation, the study should be considered a preliminary pilot investigation. Further multicenter studies with longer follow-up are needed to confirm the generalizability and clinical utility of the proposed model.

Third, we acknowledge that the exclusion of environmental and behavioral predictors—such as time spent outdoors, near-work intensity, and degree of urbanicity—is a significant limitation. Therefore, our predictions should be interpreted as reflecting baseline biological risk, which must be integrated into the patient’s lifestyle and supplemented by clinical counseling. It should be emphasized that this tool serves as a decision-support aid rather than a standalone diagnostic instrument.

## 6 Conclusion

Over the 1-year study period, premyopic children exhibited significant myopic progression, axial elongation, and subfoveal choroidal thinning, with a myopia incidence of 49.3%. We developed machine learning-based models to predict myopia onset and progression in school-aged premyopes. These models demonstrated high accuracy and identified key risk factors: lower baseline SE, parental myopia, thinner baseline SFCT, and older age collectively increased 1-year myopia risk. Our research facilitates the early identification of high-risk premyopic individuals for targeted intervention, which can improve the efficiency of myopia prevention among school-age children.

## Data Availability

The raw data supporting the conclusions of this article will be made available by the authors, without undue reservation.

## Glossary

UCVA: Uncorrected visual acuity
BCVA: Best-corrected visual acuity
IOP: Intraocular pressure
SE: Spherical equivalent
AL: Axial length
CC: Corneal curvature
SFCT: Subfoveal choroidal thickness
AL/CR: Axial length/corneal radius
SHAP: Shapley Additive exPlanations
ML: Machine learning
EPV: Events per variable
LR: Logistic regression
NB: Naïve Bayes
DT: Decision tree
RF: Random Forest
SVM: Support vector machine
ROC: Receiver operating characteristic curve
AUC: Area under the receiver operating characteristic curve
MSE: Mean squared error
MAE: Mean absolute error
RMSE: root mean squared error
R^2^: Coefficient of determination
