# Supplementary material for: Prediction of myopia onset and shift in premyopic school-aged children: a machine learning-based algorithm
Source: Front Med (Lausanne). 2025 Nov 17;12:1646277. doi: 10.3389/fmed.2025.1646277 (PMC12665658; doi:10.3389/fmed.2025.1646277)
Supplement: Supplementary file 1 [file Table_1.docx]

eTable 1 Baseline characteristics of the training set and the test set

| Variable | Train set | Test set | P |
| --- | --- | --- | --- |
| Gender, n (%) |  |  | 0.440 |
| Female | 99 (50.000) | 48 (55.814) |  |
| Male | 99 (50.000) | 38 (44.186) |  |
| Parental myopia, n (%) |  |  | 0.414 |
| 0 | 21 (10.606) | 5 (5.814) |  |
| 1 | 77 (38.889) | 37 (43.023) |  |
| 2 | 100 (50.505) | 44 (51.163) |  |
| UCVA (logMAR) | 0.004±0.049 | 0.005±0.054 | 0.906 |
| SE (D) | 0.331±0.315 | 0.334±0.311 | 0.914 |
| AL (mm) | 23.136±0.701 | 23.132±0.619 | 0.964 |
| CC (D) | 43.558±1.415 | 43.647±1.241 | 0.992 |
| AL/CR | 2.984±0.060 | 2.990±0.062 | 0.418 |
| SFCT (mm) | 307.621±33.446 | 308.105±36.901 | 0.844 |

eTable 2 Indicators were screened by univariate and multivariate logistic regression

| Variable | OR  (univariate ) | OR  lower 95%CI  (univariate ) | OR  upper 95%CI  (univariate ) | P  (univariate ) | OR  (multivariate) | OR  lower 95%CI  (multivariate) | OR  upper 95%CI  (multivariate) | P  (multivariate) |
| --- | --- | --- | --- | --- | --- | --- | --- | --- |
| SE | 0.318 | 0.279 | 0.362 | 0.000 | 0.359 | 0.306 | 0.421 | 0.000 |
| Gender | 0.922 | 0.820 | 1.038 | 0.258 |  |  |  |  |
| SFCT | 0.996 | 0.995 | 0.998 | 0.001 | 0.997 | 0.996 | 0.999 | 0.000 |
| CC | 1.019 | 0.977 | 1.063 | 0.452 |  |  |  |  |
| Age | 1.089 | 1.049 | 1.131 | 0.000 | 1.034 | 1.007 | 1.063 | 0.040 |
| AL | 1.205 | 1.111 | 1.307 | 0.000 | 0.998 | 0.938 | 1.063 | 0.965 |
| Parental myopia | 1.235 | 1.135 | 1.343 | 0.000 | 1.222 | 1.155 | 1.293 | 0.000 |
| UCVA | 3.892 | 1.184 | 12.794 | 0.060 |  |  |  |  |
| AL/CR | 55.114 | 23.196 | 130.974 | 0.000 | 1.091 | 0.483 | 2.467 | 0.859 |
